# Monitoring Urban Greenness Dynamics Using Multiple Endmember Spectral Mixture Analysis

**DOI:** 10.1371/journal.pone.0112202

**Published:** 2014-11-06

**Authors:** Muye Gan, Jinsong Deng, Xinyu Zheng, Yang Hong, Ke Wang

**Affiliations:** 1 College of Environmental and Resource Sciences, Zhejiang University, Hangzhou, Zhejiang Province, China; 2 School of Civil Engineering and Environmental Sciences and School of Meteorology, University of Oklahoma, Norman, Oklahoma, United States of America; 3 State Key Laboratory of Hydroscience and Engineering, Department of Hydraulic Engineering, Tsinghua University, Beijing, China; Instituto de Pesquisas Ecológicas, Brazil

## Abstract

Urban greenness is increasingly recognized as an essential constituent of the urban environment and can provide a range of services and enhance residents’ quality of life. Understanding the pattern of urban greenness and exploring its spatiotemporal dynamics would contribute valuable information for urban planning. In this paper, we investigated the pattern of urban greenness in Hangzhou, China, over the past two decades using time series Landsat-5 TM data obtained in 1990, 2002, and 2010. Multiple endmember spectral mixture analysis was used to derive vegetation cover fractions at the subpixel level. An RGB-vegetation fraction model, change intensity analysis and the concentric technique were integrated to reveal the detailed, spatial characteristics and the overall pattern of change in the vegetation cover fraction. Our results demonstrated the ability of multiple endmember spectral mixture analysis to accurately model the vegetation cover fraction in pixels despite the complex spectral confusion of different land cover types. The integration of multiple techniques revealed various changing patterns in urban greenness in this region. The overall vegetation cover has exhibited a drastic decrease over the past two decades, while no significant change occurred in the scenic spots that were studied. Meanwhile, a remarkable recovery of greenness was observed in the existing urban area. The increasing coverage of small green patches has played a vital role in the recovery of urban greenness. These changing patterns were more obvious during the period from 2002 to 2010 than from 1990 to 2002, and they revealed the combined effects of rapid urbanization and greening policies. This work demonstrates the usefulness of time series of vegetation cover fractions for conducting accurate and in-depth studies of the long-term trajectories of urban greenness to obtain meaningful information for sustainable urban development.

## Introduction

The rapid urbanization in China in recent decades, accompanied by the continuous increase in urban population and the unprecedented growth of cities, has put significant pressure on urban environments. This pressure has led to severe environmental issues, such as urban heat islands, air pollution, and urban flooding [1]. Moreover, improved economic conditions have lead residents to demand better living environments and a better quality of life.

Urban greenness, represented in this study by all vegetation cover in and around cities, e.g., street plantation, lawns, parks, gardens, crops, wetlands, and forests, contributes valuable ecosystem services and plays an irreplaceable role in the improvement of the urban environment [2]. Urban vegetation improves air quality [3], intercepts storm water runoff [4], reduces energy demands [5], and provides numerous psychological benefits, aesthetic views and restorative opportunities for city dwellers who may otherwise have limited exposure to natural environments [6], [7].

Rapid urbanization has resulted in significant alterations in the quality and quantity of urban greenness [8]. In a large-sample survey involving 386 European cities, green space was found to increase with the city area and slightly decline with the population density [9]. Dallimer et al. indicated that nine out of thirteen cities in England exhibited declining urban green spaces between 2000 and 2008 [10]. Infill development during urbanization is considered to be a major cause of green space loss. Similarly, a statistically significant decline in tree cover was found in many major U.S. cities, along with increased population densities and likely increased development pressure between 2003 and 2009 [11]. Unlike the declining trend reported by the above studies, green space coverage may increase with urbanization intensity. Green space coverage in the built-up areas of Chinese cities, for example, has increased steadily during the last two decades [12]. Yang et al. confirmed the greening trend in larger cities, and they note that rapid urbanization caused a dramatic turnover in vegetation cover [13]. Accurate and timely monitoring of the status of urban greenness is essential for the protection and management of urban environments. Furthermore, characterizing and understanding the trends in urban vegetation cover change provide insights into the relationship between urban greenness and urbanization and can help guide sustainable urban development [14].

As an important source of urban information, remote sensing data provide a spatially consistent coverage of large areas with both high spatial detail and high temporal frequency [15]. With the increased availability of current and historical remotely sensed data and new analytical techniques, it is now possible to quantify urban greenness in a timely and cost-effective manner [16], [17]. Despite the increasing application of high-resolution images, medium-resolution imagery (e.g., Landsat and SPOT) is still the preferred data source. Specifically, medium-resolution imagery is globally available, and it uniquely composes the only long-term, consistent digital dataset. However, commonly used detection techniques, such as the calculation of classification and vegetation indices from medium spatial resolution imagery, may be ineffective in quantifying physically fine-resolution information related to urban greenness. The urban environment is highly heterogeneous and complex, resulting in the mixed pixel problem [18], [19]. Because the status and variation of urban greenness typically occur at finer spatial scales than most moderate resolution imagery, the vegetation in a pixel can only be recorded as either present or absent with traditional hard classifiers, resulting in a loss of information on isolated vegetation and within-class variation [20], [21]. However, the status of and subtle change in urban greenness are highly related to the urban environment and significantly affect local residents' quality of life. The specific characteristics of the urban landscape require further consideration regarding the capabilities and limitations of remote sensing data and the use of appropriate analysis techniques [22], [23].

A variety of approaches has been developed to overcome the mixed pixel problem in urban landscapes. Spectral mixture analysis techniques (SMA and MESMA) [24] have been widely utilized to estimate the proportion of representative urban land cover within each pixel because of their ability to support the repeatable and accurate extraction of quantitative subpixel information with physical meaning [25], [26]. To date, a considerable number of studies have utilized this method to characterize urban compositions [18], [27], [28], map urban vegetation [16], [19], [29], and monitor urban changes [30], [31], [32], [33]. However, only a few multi-temporal studies have employed these techniques to monitor long-term changes in urban greenness [34]. Given the sharp growth in urban areas in China in terms of area and intensity, it is imperative to analyze the pattern of vegetation cover change within Chinese cities.

Taking Hangzhou, one of the most rapidly urbanizing cities in eastern China, this paper proposes a feasible and cost-effective greenness change detection method in highly-fragmented urban environment by integrating multi-date remote sensing, MESMA and GIS spatial analysis. Specifically, this paper explores the spatiotemporal dynamics and evolution of greenness in response to the rapid urbanization process in the past two decades (1990–2010) aiming to improve the understanding of the effects of urbanization on greenness patterns and provide basic information for appropriate decision-making towards urban sustainable development.

## Methods

### Study site

**Figure 1:**
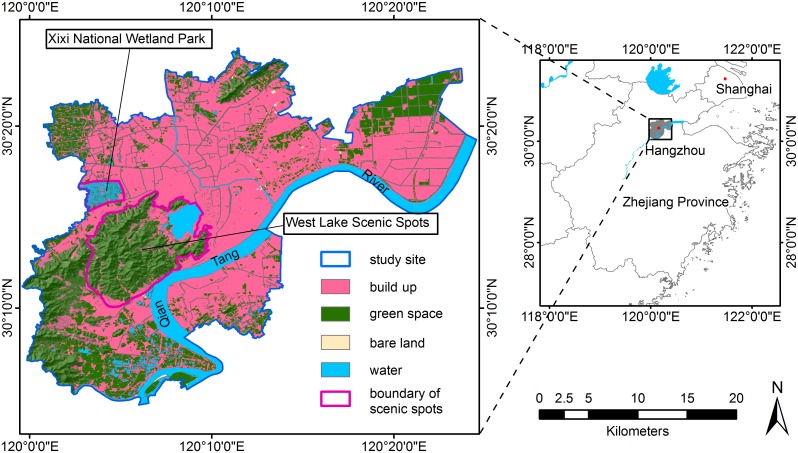
A map of the study area, i.e., Hangzhou city proper.

Hangzhou, the capital of Zhejiang Province, is located near the eastern edge of the Qiantang River in the southern Yangtze River Delta (Figure 1). This study was conducted in the area within the administrative boundaries of Hangzhou, the city proper, which covers an area of 728 km^2^ and has approximately 3.56 million registered permanent residents. At the end of 2010, the local GDP per capita was approximately 109,708 RMB Yuan (equivalent to 17,981 US dollars) [35]. As a notable international garden city, as well as a vigorous and economically competitive key city, Hangzhou has experienced significant economic development and tremendous population growth over the past decades [15]. The urbanization process has significantly modified the vegetation cover in the wetland, forest, and agriculture ecosystems in and around the city.

### Data and preprocessing

The Landsat TM images (Path: 119, Row: 39) used to conduct the study were obtained on October 8, 1990, September 23, 2002, and May 24, 2010 (i.e., a total of 20 years). The level 1T data were carefully selected from the USGS GLOVIS portal [36], with special consideration of phenology and cloudlessness. Thermal bands were excluded from the analysis. FLAASH (Fast Line-of-sight Atmospheric Analysis of Spectral Hypercubes) was used for atmospheric correction of these TM data. The study area was then extracted using the boundaries of Hangzhou.

In addition to Landsat imagery, the following ancillary data were collected for analytical purposes: (1) two historical true color aerial photographs with a 0.5-m resolution, acquired on September 26, 2002 and May 20, 2011; and (2) land-use maps derived from the National Detailed Land-use Inventory for 2010. All the auxiliary data were registered to the same projection as the TM images: WGS 84, UTM map projection Zone 50, with a root mean squared error (RMSE) of less than 15 m.

**Figure 2:**
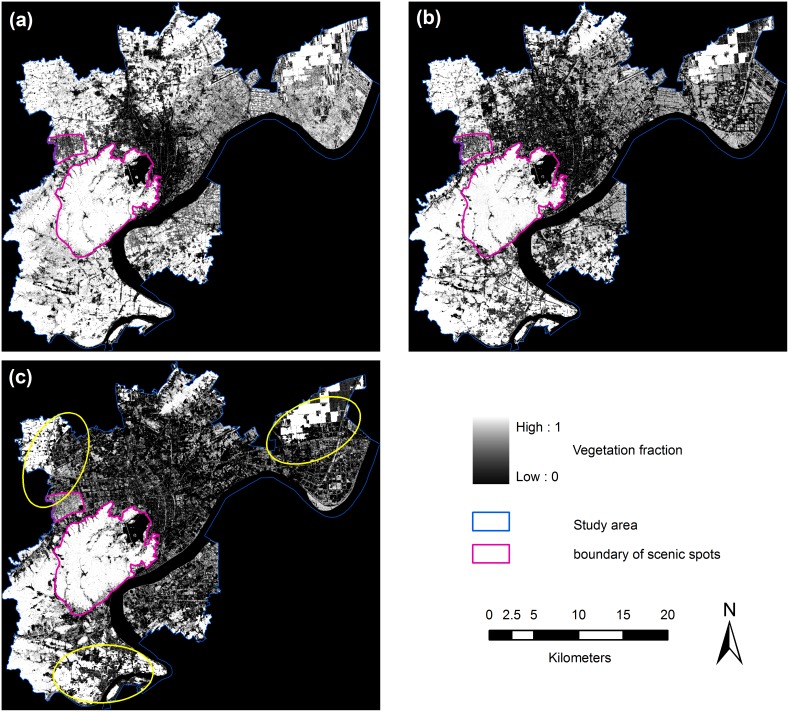
Vegetation fraction maps of Hangzhou for (a) 1990, (b) 2002, and (c) 2010. The vegetation fractions are indicated by the grayscale, where 1 represents pure vegetation (light gray) and 0 represents no vegetation (dark gray) in each pixel.

Greenness mapping requires special consideration of vegetation phenology. Generally, images from the same season with full vegetation growth are the most appropriate for detecting changes. However, because a high-quality image was not available for the late summer of 2010, a TM image acquired on May 24 was used. No significant bias in the vegetation cover was observed in late May or late September (except for cropland), according to our pre-experiment using a MODIS NDVI time series and a comparison between the fraction results from a Landsat 5 TM image acquired on May 24, 2010 and a Landsat 7 ETM+ SLC-off image acquired on September 21, 2010. To minimize the information error caused by the crop phenology, a common cropland mask was developed based on the 2010 land-use map and visual interpretation of three TM images. The common cropland pixels for the three dates were extracted, and the vegetation fraction values were set to a consistent value of 1 (Figure 2c, yellow circles). Thus, we assumed that the phenological effect of the vegetation in our study had no effect on our results during processing.

### Multiple endmember spectral mixture analysis

The SMA approach assumes that a landscape is formed from continuously varying proportions of idealized land cover types or spectrally ‘pure’ materials called endmembers. In linear SMA, the reflectance *P’* measured at pixel *i* represents a linear combination of *N* endmembers, weighted by their areal fractions *f_ki_*, within the pixel [24], [25], [33] as follows:
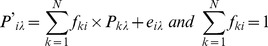
(1)where *P_kλ_* is the reflectance of endmember *k* for a specific band (*λ*) and *e_iλ_* is a residual term indicating the unmodeled portions of the spectrum. The model fit is assessed via an RMSE metric [24], [37], calculated as follows:
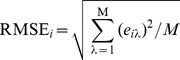
(2)where *M* is the number of bands [38]. The standard SMA has two major limitations. First, only one spectrum is allowed for each endmember, i.e., every image pixel is interpreted using the same endmember spectra. Therefore, the method is unable to account for within-class spectral variability. This is particularly problematic in Chinese cities where a large variation of material spectra exists due to rapid development and ubiquitous urban reconstruction. Second, the standard SMA is most often implemented with a fixed number of endmembers for the entire image, regardless of whether the ground components represented by the endmembers are present in the pixel. This may result in the decreased accuracy of the estimated fractions [18], [25], [39]. MESMA, which is an extension of the standard SMA approach, allows the number and type of endmembers to vary on a per pixel basis to better represent the spectral variability of land cover; thus, it is more suitable for urban landscapes [25], [27].

In this study, MESMA was conducted according to the following steps: (1) endmember selection, (2) spectral unmixing modeling, and (3) accuracy assessment. These steps are each detailed in turn. MESMA was implemented using VIPER Tools Version 1.5, which is a plug-in to the ENVI software package [40].

#### Endmember selection

The careful selection of representative endmembers is essential to all applications of SMA [20], [41], [42]. In this study, the endmembers were extracted from the 1990, 2002, and 2010 Landsat TM images. When building a spectral library for MESMA, the number of spectra needed for adequately representing the spectral variation in the materials and for attaining computational efficiency should be considered [25], [43], [44]. Therefore, the endmembers were organized into five groups: vegetation, impervious surfaces, soil, water, and shade. Each group included several subsets, e.g., vegetation (urban forest, primary forest, grass, and crops), impervious surfaces (urban center and suburbs), soil (mine soil, farmland soil, and construction sites), and water (lakes and rivers). An endmember for shade (zero value in all bands) was included in all of the models [41], [45].

First, the spectrally “pure” pixels were collected from the image based on the pixel purity index (PPI) [46] and high-resolution aerial photographs to build a candidate spectral library for each endmember. Pixels with high PPI scores, representing pure land cover in the high-resolution aerial photograph and having reasonable spectral signatures, were chosen as candidate spectra and grouped into the corresponding subclasses. The representative spectra for each subclass were then selected from the candidate spectra using the endmember average root mean squared error (EAR) [41], count-based endmember selection (CoB) [47], and minimum average spectral angle (MASA) [38]. The optimal set of spectra for each endmember was iteratively selected by adding spectra with a high CoB index, low EAR, or low MASA to the library and assessing the model performance using RMSE images and a visual comparison with the high-resolution aerial photographs [16]. Ultimately, libraries with 47, 53, and 69 spectra were used for the 1990, 2002, and 2010 images, respectively, to run the unmixing models.

#### Spectral unmixing modeling

Previous research has shown that natural systems are usually best modeled by two endmembers, while disturbed regions require three and urban landscapes require four [48]. Therefore, two-endmember models with all the spectra were first applied to map each pixel in the image (Table 1). Then, only spectra that effectively represented the pure land cover within the urban area in the two-endmember models were retained to run three- and four-endmember models. Intra-class spectral mixtures were only considered for impervious surfaces [48]. Every model was evaluated for every pixel in the image. Constraints were determined based on iterative experimentation and the findings of previous studies [37], [41], [45]. The non-shade fractions were confined to the range of −0.05 to 1.05, the maximum allowable shade fraction was set to 0.5, and a maximum RMSE of 2.5% in reflectance was applied.

**Table 1:** Combinations of endmember models by land cover class.

2-endmember	3-endmember	4-endmember
Imp+SHD	Imp_a_+Imp_b_+SHD	Imp+V+S+SHD
V+SHD	Imp+V+SHD	Imp+V+W+SHD
S+SHD	Imp+S+SHD	S+V+W+SHD
W+SHD	Imp+W+SHD	Imp_a_+Imp_b_+V+SHD
	V+S+SHD	Imp_a_+Imp_b_+S+SHD
	V+W+SHD	Imp_a_+Imp_b_+W+SHD
	S+W+SHD	

V refers to green vegetation, Imp to impervious surface, S to soil, W to water, and SHD to shade. Endmembers with subscripts (a, b) were used multiple times. The same endmember spectra were not used in one combination.

After the models with the different levels of spectral complexity (i.e., two-, three-, and four-endmember models) were applied, the model with the lowest RMSE at each level was compared to determine the best model for each pixel. Generally, a lower RMSE in a more complex model does not necessarily indicate better modeling of the true land cover component [28], [49]. In addition, we noticed that the introduction of a water endmember significantly improved the model performance in the water-containing and water-edge pixels (e.g., wetlands), but it also caused a mis-unmixing between water and shade classes in pixels affected by topographic shadowing within mountainous regions [50]. Based on previous findings, simpler endmember models are preferred over more complex endmember models, except in cases in which adding an endmember significantly improves the RMSE beyond a specified threshold [30], [47], [50]. The RMSE threshold value between the two- and three-endmember models was 0.2% for the reflectance, the RMSE threshold between the three- and four-endmember models was 0.4%, and the RMSE threshold between the two- and four-endmember models was 0.6%. When a water endmember was used, the corresponding threshold values were set to 0.7%, 0.4% and 1.1%, respectively, for the reflectance.

The fractions produced by the optimal models were then shade-normalized [24], rescaled [26], and combined to generate fractional maps of each component. Vegetation cover fraction (VCF) maps were retained to investigate the vegetation cover change.

#### Accuracy assessment

Evaluating vegetation fraction estimates can be challenging due to the difficulty of obtaining reference data, especially for historical datasets. Because of the lack of high-quality reference images from the 1990s, accuracy assessments for only two dates were performed using the aerial photographs acquired in 2002 and 2011 for the relevant TM images. We assumed that the result of the 1990 fraction image would have acceptable accuracy if the vegetation fraction images in 2002 and 2010 were reasonably accurate. The lack of an accuracy assessment for the 1990 fraction image is a limitation of this study.

Two hundred samples were used to assess the modeled VCF after removing the samples with apparent land cover changes. Each sample had a window size of 3×3 pixels to reduce the impact of geometric errors between the aerial photographs and the TM images [27], [51]. The ‘true’ proportion of vegetation was then calculated by dividing the area of vegetation cover measured through visually interpreting the aerial photograph by the total sampling area (i.e., 8,100 m^2^). The accuracy of the vegetation cover fraction was assessed using three metrics: the coefficient of determination (R^2^), the root mean squared error (RMSE), and the systematic error (SE) [52] as follows:
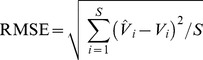
(3)

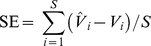
(4)where 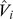 is the modeled fractional value of vegetation measured at sample *i*, *V_i_* is the reference fractional value, and *S* is the number of samples. The RMSE is a measure of the overall estimation accuracy for all samples, and the SE is a measure of the effects of systematic errors (e.g., overestimation or underestimation) [53].

### RGB-vegetation fraction model

An RGB-vegetation fraction model based on additive color theory was applied to the multi-temporal vegetation fraction images to interpret change events in our study area. Using this technique, multiple dates of vegetation fraction images could be viewed at one time, and the changes were highlighted. Vegetation fraction images in 1990, 2002, and 2010 were placed chronologically by red, green, and blue colors, respectively. Any combination of primary colors of similar brightness produces a complementary color that can identify the direction and magnitude of the vegetation cover change.

### Change intensity analysis

To study the spatial distribution of the vegetation change intensity, fraction change images from 1990 to 2002 (T1) and from 2002 to 2010 (T2) were produced by subtracting two sequential fraction images. The intensity of the fraction change was divided into 4 intensity categories: extreme decrease (−1 to −0.5), moderate decrease (−0.5 to −0.2), moderate increase (0.2 to 0.5) and extreme increase (0.5 to 1). The relationship between the change intensity and the distance to the urban center was analyzed by counting the percentage of pixels of a certain intensity category at a certain distance.

### Concentric analysis

**Figure 3:**
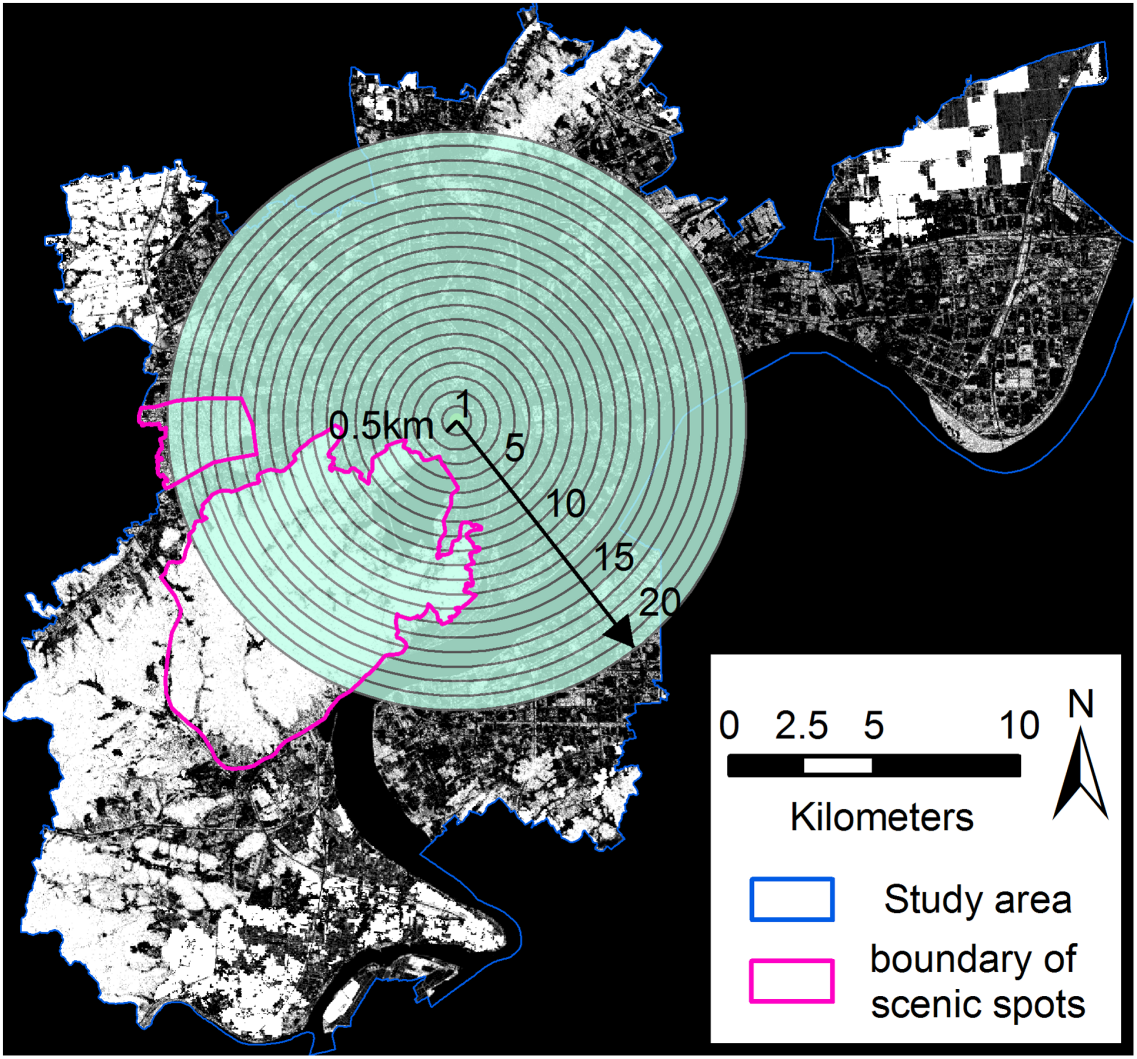
The layout of the concentric belts. Twenty concentric belts radiating from the city center, where each belt spans 0.5 km.

A concentric analysis technique was used to obtain insight into the relationship between urban vegetation status and urban expansion. Twenty concentric belts were identified, radiating from the urban center to the fringe (Figure 3). Each belt spanned 0.5 km, approximately 16 pixels in the TM image, which is approximately two times the minimum resolution of assessment suggested by Powell et al. [18]. To account for the multi-nuclei expansion pattern in Hangzhou [54], we only applied the concentric analysis to the main urban area rather than to the entire study area. Furthermore, two famous tourist attractions, the West Lake Scenic Spots (WLSS) and the Xixi National Wetland Park (XNWP), were excluded from the concentric analysis because they were subject to a different land use policy than the rest of the study area.

MESMA provided subpixel information with physical meaning, where a fraction value of 1 represented full coverage of vegetation within a pixel. The vegetation fraction images were reclassified into 4 density categories: low coverage (0 to 0.2), middle coverage (0.2 to 0.5), middle-high coverage (0.5 to 0.8), and high coverage (0.8 to 1). The overall average vegetation fraction and the area percentages of the pixels within the 4 density categories were calculated for each belt to estimate the general pattern of urban greenness.

## Results

### Accuracy assessment

**Figure 4:**
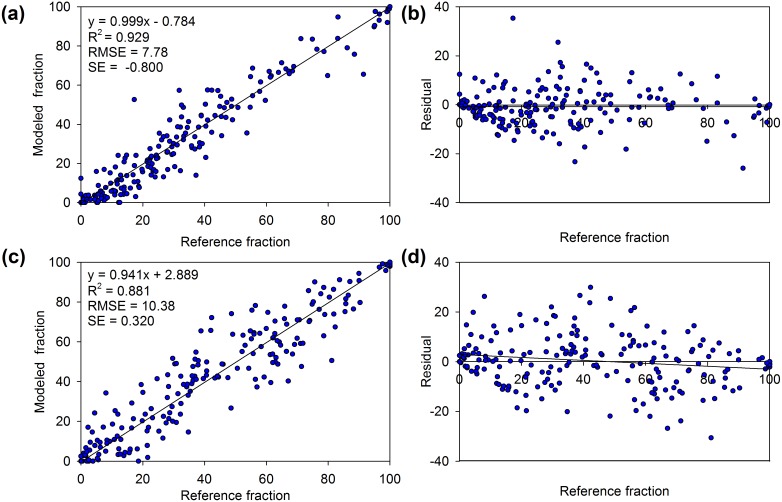
Comparisons between the reference and modeled fractions. (**a**) Vegetation fraction scatter plot for 2010, (**b**) vegetation fraction residuals for 2010, (**c**) vegetation fraction scatter plot for 2002, and (**d**) vegetation fraction residuals for 2002. Perfect agreement, represented by the 1∶1 line, is displayed in the scatter plots. The best-fit line is displayed in the residual plots to indicate the general trends of overestimation and underestimation.

The R^2^, RMSE, and SE were calculated for the 2002 and 2010 vegetation fraction images to evaluate the modeled results quantitatively. Scatter plots of the accuracy assessment results are shown in Figure 4. The RMSE was 7.78% for the 2010 image and 10.38% for the 2002 image. The R^2^ values were relatively high, ranging from 0.929 to 0.881, indicating a good fit between the ‘true’ fraction and the fraction derived from MESMA.

The analysis of the residuals, in terms of the SE (−0.800% for 2010 and 0.320% for 2002), indicated that there was no significant underestimation or overestimation relative to the reference fraction.

### Spatiotemporal analysis of urban greenness change

To construct vegetation cover images for corresponding years, the amount of vegetation in each pixel was represented by the assigned gray value. Brighter areas signify higher proportions of vegetation, and darker areas signify lower proportions of vegetation.

Overall, the urban greenness of Hangzhou has decreased steadily over the past two decades (Figures 2a, b, and c). In 1990, most of the high-density green space was distributed in natural land-use areas (i.e., agricultural areas, primary forests, and wetlands) around the built-up area, and the vegetation cover within the city was relatively sparse. Along with the urban expansion, an obvious loss of greenness occurred in the urban fringe and the suburban area. The urban greenness became more fragmented and isolated, except in mountainous areas and in the remaining cropland. The total percentage of the vegetated area of Hangzhou decreased greatly from 64.61% to 42.76% (Table 2), especially in T2, nearly twice the rate observed in T1.

**Table 2:** Total percent vegetated area and changes from 1990 to 2010 for the study area and the two scenic spots.

	1990	2002	2010	1990–2002(per year)	1990–2002(per year)
TPVA	64.61%	54.51%	42.76%	−0.84%	−1.53%
TPVA in XNWP	52.39%	54.44%	58.92%	0.17%	0.58%
TPVA in WLSS	85.51%	84.70%	83.13%	−0.07%	−0.20%

TPVA is the total percent of vegetated area, XNWP is the Xixi National Wetland Park, and WLSS is the West Lake Scenic Spots.

**Figure 5:**
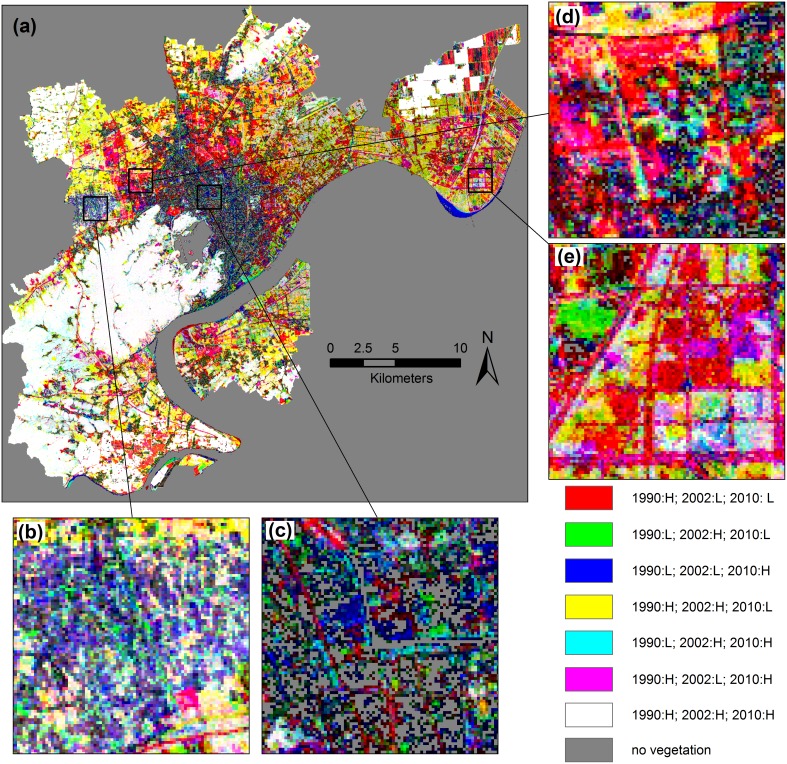
RGB color composite using vegetation fraction maps of 1990 (R), 2002 (G) and 2010 (B). The typical examples demonstrated are (a) the study area, (b) Xixi National Wetland Park, (c) the urban center, (d) residential communities and (e) the Xiasha suburban college town. Colors for the typical compositions of the vegetation fractions on the three dates are illustrated. H represents high vegetation fraction and L represents low vegetation fraction.

Using the RGB-vegetation fraction model, distinctly different patterns were demonstrated among the various segments of the urban area (Figure 5). The core built-up area exhibited an increasing percentage of vegetation cover in T1 and T2, dominated by cyan and blue colors (Figure 5c). The other colors, such as red, yellow and green, revealed a complex reconfiguration of urban greenness in the core built-up area over the past two decades due to urban reconstruction and the urban greening project. In contrast, the urban peripheral areas showed dramatic declines in vegetation cover in the two periods. Vast fields of red pixels were observed near the core built-up area, indicating vegetation loss occurred during the urban expansion in T1 (Figure 5b). Two large blocks of vegetation reduction occurred in the eastern and southern parts of the city due to the construction of two new subcities (Xiasa and Jiangnan). Furthermore, a more severe decrease in the greenness in T2 could be confirmed by the widespread yellow color around the existing built-up areas in the main city and in the two subcities. In Xiasa, a suburban college town, road networks and communities constructed in T1 were depicted in red (Figure 5e). The remaining vegetation along the road networks was replaced by new construction in T2, indicated by yellow. Meanwhile, some of the roads and communities represented by magenta suggest vegetation recovery in the form of road and community greening after 2002. In most WLSS regions, the vegetation coverage remained consistently high, as indicated in white. However, a much more complex color composition of blue, green, purple and magenta was observed in XNWP and reflects a complex but overall subtle increase in wetland vegetation coverage over the past two decades.

**Figure 6:**
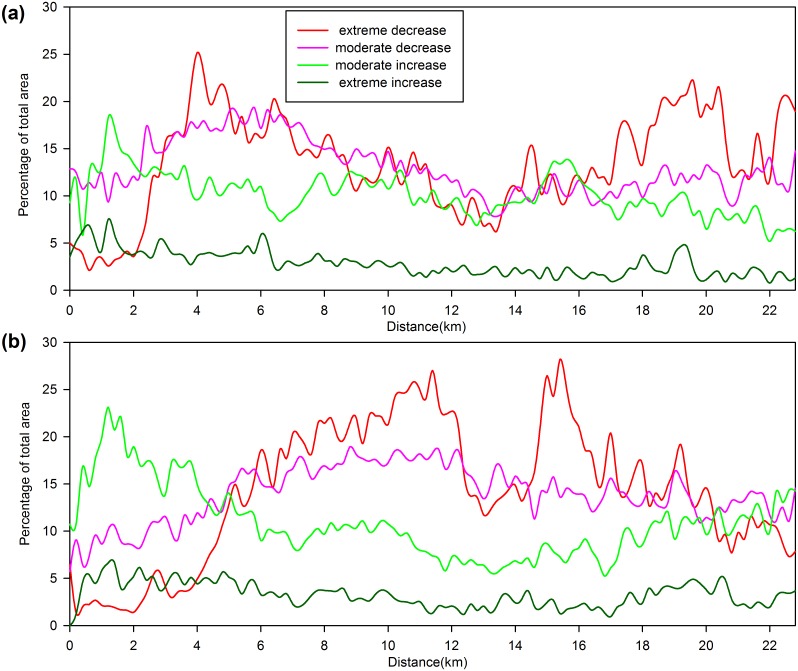
Spatial characteristics of the vegetation fraction change from the urban center over the two periods. (a) 1990–2002 and (b) 2002–2010. The vegetation change categories are shown by the different colors in the legend.

A clear spatial disparity in vegetation coverage change intensity was found (Figure 6) by analyzing the relationships between the change intensity and the distance to the urban center. In T1, the vegetation change pattern was dominated by a moderate increase within 2 km. At 2 km, the extreme decrease soared to over 20% and became the major change process. Two peaks appeared at approximately 4 km and 19 km, reflecting the vegetation loss due to the expansion of the core built-up area and construction of the new subcities. In T2, a greater intensity and extent of moderate increases was observed; this suggests that vegetation coverage more vigorously recovered in the core built-up area. However, a more severe extreme decrease occurred in the outer region of the core built-up area and subcities and peaked at approximately 11 km and 15 km. Therefore, the hotspot of the vegetation decline moved to the suburban area between the core built-up area and the subcities. The conversion, both negative and positive, was much more significant in the later decade. A pattern similar to the class of the extreme decreases could be found in the class of moderate decreases, while the class of extreme increases remained consistently low and showed no distinct pattern related to distance in either period.

### General pattern of urban greenness accompanied by urban expansion

Twenty concentric belts radiating from the city center to the fringe were analyzed to provide insights into the spatial patterns of urban greenness change within and around the city during the two periods. In 1990, the average vegetation fraction remained low in belts 1–5 but increased significantly in belt 6; this highlights the difference in vegetation cover between the core built-up area and the suburbs. Because of urban expansion and sprawl, the boundary between the core built-up area and the suburbs was extrapolated to location near belt 9 in 2002. Meanwhile, a slight increase was detected for belts 1–5, indicating an improvement in the vegetation cover in the existing urban area. By 2010, a more obvious increase occurred in belts 1–9, along with a more severe decrease in belts 10–20. Additionally, the correlation between the vegetation abundance and the distance from the city center became weaker. These changes occurred because nearly all the regions within the twenty belts were highly urbanized, and only sparse native vegetation remained in belts 16–20. With the expansion and sprawl of built-up areas, urban greenness declined sharply in the urban fringe and the suburbs. A subsequent recovery of the vegetation cover occurred in the existing urban area, along with a decrease in the outer belts. This reflects the main process of conversion from natural vegetation to artificial urban greening because of urban expansion.

**Figure 7:**
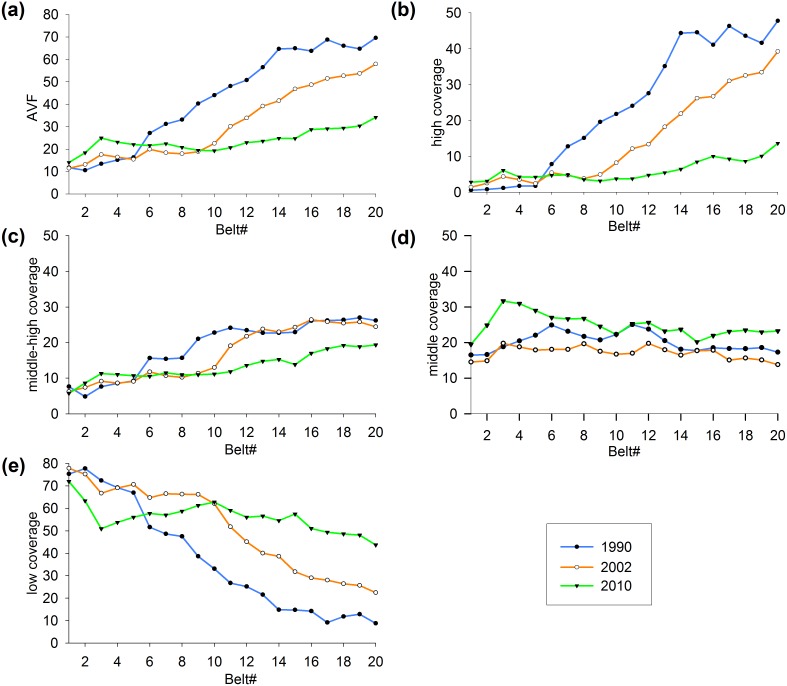
Concentric vegetation coverage analysis in each belt for each year. (a) Average vegetation fraction (AVF), (b) percentage of area of high coverage pixels, (c) percentage of area of middle-high coverage pixels, (d) percentage of area of middle coverage pixels, and (e) percentage of area of low coverage pixels.

Urban greenness in the existing urban area contributes valuable ecosystem services which are related closely with the daily life of the local residents [55]. The lowest vegetation fraction was found in 1990 (Figure 7a: belts 1–5, 1990). Since then, the vegetation cover continued to increase in the older neighborhoods over the past two decades (belts 1–5 in T1 and belts 1–9 in T2), indicating sustained improvement in the urban environment can be a component of the urbanization. In 2010, the average vegetation fraction of the urban area was nearly double that in 1990. In addition, an obvious increase in the middle cover pixels occurred in belts 1–10, especially in belts 1–5 (Figure 7d), along with a decrease in the low cover pixels (Figure 7e). The result of change intensity analysis also showed that the changing pattern in urban center was dominated by moderate increase. These characteristics of vegetation fraction within a pixel further indicated those small green patches with size smaller than the pixel size of the Landsat data has mainly contributed to the increase of green space in existing urban area.

## Discussion

### MESMA and integration of the multiple analysis technique

The accuracy of the information acquired on vegetation cover has a direct influence on how well the trends and processes of greenness change are understood, especially for a diverse urban area. Urban greenness is generally highly fragmented in Chinese cities, resulting in a large number of small patches. Li et al. indicated that 86% of the patches of urban greenness were smaller than 900 m^2^, and 37% were smaller than 100 m^2^ in their study area [56]. Likewise, our study highlights the importance of the increasing area of small green patches, which may be difficult to detect by traditional classification techniques. An analysis based on directly classifying medium-resolution data would lead to an underestimation of the amount of urban greenness, which is crucial to urban areas with generally limited amounts of vegetation cover. This study demonstrated the advantage of MESMA in providing accurate and reliable subpixel information with physical meaning. However, this method still failed to acquire accurate information on the species and structure of urban greenness in multispectral images. Moreover, the method was unable to resolve nonlinear mixing effects, and it generally needed more supervision in the unmixing process. By integrating RGB-vegetation fraction model, the change intensity analysis and concentric analysis, detailed information on the direction, magnitude, location and pattern of the variation of urban greenness were able to be visually and quantitatively analyzed. These information have improved our understanding of how urban greenness evolved under rapid urbanization. The strength of MESMA is its ability to represent a wide variety of surface reflectance types as simple combinations of endmember abundances. Furthermore, because of the global availability and rich historical data archive of the Landsat data, analysis based on these techniques can be easily expanded to other cities around the world.

### Greenness change in response to urbanization and greening policies

Despite the proven benefits of urban green spaces [3], [4], [5], [6], [7], widespread losses of these spaces have been reported in American and European cities [9], [11]. In our study area, one-third of the vegetation cover that was present in 1990 was gradually lost over these two decades. The RGB-vegetation fraction model showed that most of the vegetation loss occurred in the urban fringe or in the new subcities. This trend agrees with the regional decline in vegetation cover caused by urbanization, as observed by Sun et al. [57]. In contrast to the overall decline throughout the study area, both the RGB-vegetation fraction model and concentric analysis demonstrated a consistent increase in urban greenness in the existing urban area. This confirmed the finding that Chinese cities have undergone a steady increase in urban greenness in the built-up areas [12]. We further demonstrated the contribution to the recovery of urban greenness by the increasing area of small green patches. The vegetation cover of the scenic areas remained relatively stable, even with a slight increase in XNWP, which reflects the effect of a scenic protection policy and an ecological renewal project. The changing pattern of greenness in the study area from 1990 to 2010 was a response to the combined effects of rapid urbanization and greening policies. Because of China’s unique institutional character, cities have developed into modern metropolitan areas in a much different way than other regions. The government plays a key role in guiding urban planning. Greenness was very limited in the urban area in 1990, and most of the greenness was located in the West Lake Scenic Spots. This uneven distribution limited the access of urban residents to the ecological benefits of urban green space. The high degree of urbanization in the city center and the rapid growth of urban population exacerbated the demand for urban greenness but also placed limits on its development. The implementation of new urbanization strategies since 2000, such as “leapfrog development along the Qiantang River” and “crossing the Qiantang River and developing southward”, triggered eastward sprawl and the construction of two subcities [54], resulting in the use of more space to reduce the population and building density and the availability of space for the creation and development of urban green space system. A green network system called “Two rings, Two axles, Six ecological zones” and a series of greening projects, e.g., the West Lake comprehensive protection project, the Grand Canal comprehensive protection project, and the Xixi wetland comprehensive protection project, have been conducted. These projects contributed to the accelerated recovery of urban greenness from 2002 to 2010 within the city. Meanwhile, innovative efforts such as retrofitting green space alongside formerly dilapidated canals, underneath and alongside main roads and railway lines were done to activate neglected spaces to restore lost green space [58]. In addition, the urban greening management regulation of Hangzhou set the minimum greening cover for new residential area to 30%. These factors explain the remarkable increase in the number of middle cover pixels.

## Conclusions

Acquiring accurate and area-wide information remains a challenge in urban environments due to the extremely heterogeneous spatial patterns within cities. Subpixel methods can overcome this problem and provide valuable quantitative information. In this paper, we investigated the patterns of change in urban greenness in Hangzhou, China, over the past two decades using Landsat imagery from 1990, 2002, and 2010. Multiple endmember spectral mixture analysis (MESMA) was used to derive vegetation cover fractions (VCFs) for various segments of the urban area. Despite the complex spectral confusion of different land cover types in the urban area, MESMA could provide accurate and reliable subpixel information of the vegetation cover fraction. An RGB-vegetation fraction model, change intensity analysis and the concentric technique were integrated to characterize the detailed, spatial characteristics and overall pattern of the urban greenness change over time.

The study area has experienced a drastic loss of urban greenness. Nearly one-third of the vegetation coverage that was present in 1990 was gradually lost over the past two decades. Most of this loss is located in the urban fringe and suburban areas due to urban expansion and the construction of new subcities. No significant change occurred in scenic spots, except for a recovery of vegetation coverage in the Xixi National Wetland Park from 2002 to 2010. Meanwhile, a remarkable recovery in the vegetation coverage occurred in the existing urban area that was mainly attributed to the continuous increase in small green patches. These changing patterns were more obvious from 2002 to 2010 than from 1990 to 2002. The characteristics of the changing pattern of urban greenness over time and in different parts of the urban environment reveal the combined effects of rapid urbanization and greening policies.

This study demonstrated the unique role of medium-resolution satellite images in the longitudinal analysis of the evolution of urban greenness. Subpixel methods provided abundant meaningful information, and more analysis techniques could be developed based on such continuous information. Furthermore, regional comparisons of cities with different economic and natural conditions should be conducted using these techniques to investigate the relationships between urban greenness and urbanization.

## References

[1] Grimm NB, Faeth SH, Golubiewski NE, Redman CL, Wu JG, et al (2008) Global change and the ecology of cities. Science 319: 756–760. 18258902 10.1126/science.1150195

[2] Robinson SL, Lundholm JT (2012) Ecosystem services provided by urban spontaneous vegetation. Urban Ecosystems 15: 545–557.

[3] Davies RG, Barbosa O, Fuller RA, Tratalos J, Burke N, et al (2008) City-wide relationships between green spaces, urban land use and topography. Urban Ecosystems 11: 269–287.

[4] Walsh CJ, Fletcher TD, Burns MJ (2012) Urban Stormwater Runoff: A New Class of Environmental Flow Problem. PLoS ONE 7: e45814. 23029257 10.1371/journal.pone.0045814 PMC3446928

[5] Akbari H, Pomerantz M, Taha H (2001) Cool surfaces and shade trees to reduce energy use and improve air quality in urban areas. Solar Energy 70: 295–310.

[6] Donovan GH, Butry DT, Michael YL, Prestemon JP, Liebhold AM, et al (2013) The Relationship Between Trees and Human Health: Evidence from the Spread of the Emerald Ash Borer. American Journal of Preventive Medicine 44: 139–145. 23332329 10.1016/j.amepre.2012.09.066

[7] Everson-Rose SA, Lewis TT (2004) Psychosocial factors and cardiovascular diseases. Annual Review of Public Health 26: 469–500. 10.1146/annurev.publhealth.26.021304.144542 15760298

[8] Tan PY, Wang J, Sia A (2013) Perspectives on five decades of the urban greening of Singapore. Cities 32: 24–32.

[9] Fuller RA, Gaston KJ (2009) The scaling of green space coverage in European cities. Biology Letters 5: 352–355. 19324636 10.1098/rsbl.2009.0010 PMC2679924

[10] Dallimer M, Tang ZY, Bibby PR, Brindley P, Gaston KJ, et al (2011) Temporal changes in greenspace in a highly urbanized region. Biology Letters 7: 763–766. 21429910 10.1098/rsbl.2011.0025 PMC3169039

[11] Nowak DJ, Greenfield EJ (2012) Tree and impervious cover change in US cities. Urban Forestry & Urban Greening 11: 21–30.

[12] Zhao JJ, Chen SB, Jiang B, Ren Y, Wang H, et al (2013) Temporal trend of green space coverage in China and its relationship with urbanization over the last two decades. Science of the Total Environment 442: 455–465. 23186616 10.1016/j.scitotenv.2012.10.014

[13] Yang J, Huang C, Zhang Z, Wang L (2013) The temporal trend of urban green coverage in major Chinese cities between 1990 and 2010. Urban Forestry & Urban Greening.

[14] Zhou XL, Wang YC (2011) Spatial-temporal dynamics of urban green space in response to rapid urbanization and greening policies. Landscape and Urban Planning 100: 268–277.

[15] Deng JS, Wang K, Hong Y, Qi JG (2009) Spatio-temporal dynamics and evolution of land use change and landscape pattern in response to rapid urbanization. Landscape and Urban Planning 92: 187–198.

[16] Liu T, Yang XJ (2013) Mapping vegetation in an urban area with stratified classification and multiple endmember spectral mixture analysis. Remote Sensing of Environment 133: 251–264.

[17] He C, Convertino M, Feng Z, Zhang S (2013) Using LiDAR Data to Measure the 3D Green Biomass of Beijing Urban Forest in China. PLoS ONE 8: e75920. 24146792 10.1371/journal.pone.0075920 PMC3795711

[18] Powell RL, Roberts DA, Dennison PE, Hess LL (2007) Sub-pixel mapping of urban land cover using multiple endmember spectral mixture analysis: Manaus, Brazil. Remote Sensing of Environment 106: 253–267.

[19] Small C (2001) Estimation of urban vegetation abundance by spectral mixture analysis. International Journal of Remote Sensing 22: 1305–1334.

[20] Myint SW (2006) Urban vegetation mapping using sub-pixel analysis and expert system rules: A critical approach. International Journal of Remote Sensing 27: 2645–2665.

[21] Foody GM, Boyd DS (1999) Detection of partial land cover change associated with the migration of inter-class transitional zones. International Journal of Remote Sensing 20: 2723–2740.

[22] Herold M, Couclelis H, Clarke KC (2005) The role of spatial metrics in the analysis and modeling of urban land use change. Computers, Environment and Urban Systems 29: 369–399.

[23] Patino JE, Duque JC (2013) A review of regional science applications of satellite remote sensing in urban settings. Computers, Environment and Urban Systems 37: 1–17.

[24] Adams JB, Smith MO, Gillespie AR (1993) Imaging spectroscopy: Interpretation based on spectral mixture analysis. In: CM P, P E, ∧editors. Remote geochemical analysis: elemental and mineralogical composition: New York: Cambridge Univ. pp. 145–166.

[25] Roberts DA, Gardner M, Church R, Ustin S, Scheer G, et al (1998) Mapping chaparral in the Santa Monica Mountains using multiple endmember spectral mixture models. Remote Sensing of Environment 65: 267–279.

[26] Smith MO, Ustin SL, Adams JB, Gillespie AR (1990) Vegetation in deserts: I. A regional measure of abundance from multispectral images. Remote Sensing of Environment 31: 1–26.

[27] Lu DS, Weng QH (2004) Spectral mixture analysis of the urban landscape in Indianapolis with landsat ETM plus imagery. Photogrammetric Engineering & Remote Sensing 70: 1053–1062.

[28] Rashed T, Weeks JR, Roberts D, Rogan J, Powell R (2003) Measuring the physical composition of urban morphology using multiple endmember spectral mixture models. Photogrammetric Engineering & Remote Sensing 69: 1011–1020.

[29] Small C, Lu JWT (2006) Estimation and vicarious validation of urban vegetation abundance by spectral mixture analysis. Remote Sensing of Environment 100: 441–456.

[30] Michishita R, Jiang Z, Xu B (2012) Monitoring two decades of urbanization in the Poyang Lake area, China through spectral unmixing. Remote Sensing of Environment 117: 3–18.

[31] Powell RL, Roberts DA (2010) Characterizing urban land-cover change in Rondônia, Brazil: 1985 to 2000. Journal of Latin American Geography 9: 183–211.

[32] Weng QH, Lu DS (2009) Landscape as a continuum: an examination of the urban landscape structures and dynamics of Indianapolis City, 1991–2000, by using satellite images. International Journal of Remote Sensing 30: 2547–2577.

[33] Rashed T, Weeks JR, Stow D, Fugate D (2005) Measuring temporal compositions of urban morphology through spectral mixture analysis: toward a soft approach to change analysis in crowded cities. International Journal of Remote Sensing 26: 699–718.

[34] Tang JM, Chen F, Schwartz SS (2012) Assessing spatiotemporal variations of greenness in the Baltimore-Washington corridor area. Landscape and Urban Planning 105: 296–306.

[35] Hangzhou almanac 2011 (2012) Beijing: Fangzhi Press. 546 p.

[36] USGS Global Visualization Viewer. Available: http://glovis.usgs.gov/. Accessed 2013 Oct 17.

[37] Roberts DA, Batista G, Pereira J, Waller E, Nelson B (1999) Change Identification Using Multitemporal Spectral Mixture Analysis: Applications in Eastern Amazonia. In: Lunetta R, Elvidge C, ∧editors. London: Taylor & Francis. pp. 318, 16.

[38] Dennison PE, Halligan KQ, Roberts DA (2004) A comparison of error metrics and constraints for multiple endmember spectral mixture analysis and spectral angle mapper. Remote Sensing of Environment 93: 359–367.

[39] Song CH (2005) Spectral mixture analysis for subpixel vegetation fractions in the urban environment: How to incorporate endmember variability? Remote Sensing of Environment 95: 248–263.

[40] Roberts DA, Halligan K, Dennison PE (2007) VIPER Tools user manual (Version1.5) University of California at Santa Barbar a, 91P. Available: http://www.vipertools.org/. Accessed 2013 Aug 28.

[41] Dennison PE, Roberts DA (2003) Endmember selection for multiple endmember spectral mixture analysis using endmember average RMSE. Remote Sensing of Environment 87: 123–135.

[42] Tompkins S, Mustard JF, Pieters CM, Forsyth DW (1997) Optimization of endmembers for spectral mixture analysis. Remote Sensing of Environment 59: 472–489.

[43] Okin GS, Roberts DA, Murray B, Okin WJ (2001) Practical limits on hyperspectral vegetation discrimination in arid and semiarid environments. Remote Sensing of Environment 77: 212–225.

[44] Somers B, Asner GP, Tits L, Coppin P (2011) Endmember variability in Spectral Mixture Analysis: A review. Remote Sensing of Environment 115: 1603–1616.

[45] Powell R (2011) Characterizing Urban Subpixel Composition Using Spectral Mixture Analysis. In: Yang X, edito. Urban Remote Sensing: Monitoring, Synthesis and Modeling in the Urban Environment. chichester,UK: Wiley-blackwell. pp. 111–128.

[46] Boardman JW, Kruse FA, Green RO (1995) Mapping target signatures via partial unmixing of AVIRIS data. In AVIRIS Airborne Geoscience Workshop Proceedings. Pasadena.

[47] Roberts DA, Dennison PE, Gardner ME, Hetzel Y, Ustin SL, et al (2003) Evaluation of the potential of Hyperion for fire danger assessment by comparison to the Airborne Visible/Infrared Imaging Spectrometer. IEEE Transactions on Geoscience & Remote Sensing 41: 1297–1310.

[48] Powell RL, Roberts DA (2008) Characterizing Variability of the Urban Physical Environment for a Suite of Cities in Rondonia, Brazil. Earth Interactions 12.

[49] Dennison PE, Roberts DA (2003) The effects of vegetation phenology on endmember selection and species mapping in southern California chaparral. Remote Sensing of Environment 87: 295–309.

[50] Michishita R, Gong P, Xu B (2012) Spectral mixture analysis for bi-sensor wetland mapping using Landsat TM and Terra MODIS data. International Journal of Remote Sensing 33: 3373–3401.

[51] Lu DS, Moran E, Hetrick S (2011) Detection of impervious surface change with multitemporal Landsat images in an urban-rural frontier. ISPRS Journal of Photogrammetry and Remote Sensing 66: 298–306. 21552379 10.1016/j.isprsjprs.2010.10.010 PMC3085910

[52] Wu CS, Yuan F (2007) Seasonal sensitivity analysis of impervious surface estimation with satellite imagery. Photogrammetric Engineering & Remote Sensing 73: 1393–1401.

[53] Wu CS (2004) Normalized spectral mixture analysis for monitoring urban composition using ETM plus imagery. Remote Sensing of Environment 93: 480–492.

[54] Wu K, Zhang H (2012) Land use dynamics, built-up land expansion patterns, and driving forces analysis of the fast-growing Hangzhou metropolitan area, eastern China (1978–2008). Applied Geography 34: 137–145.

[55] Jim CY, Chen WY (2009) Ecosystem services and valuation of urban forests in China. Cities 26: 187–194.

[56] Li XM, Zhou WQ, Ouyang ZY (2013) Relationship between land surface temperature and spatial pattern of greenspace: What are the effects of spatial resolution? Landscape and Urban Planning 114: 1–8.

[57] Sun J, Wang X, Chen A, Ma Y, Cui M, et al (2011) NDVI indicated characteristics of vegetation cover change in China’s metropolises over the last three decades. Environmental monitoring and assessment 179: 1–14. 20853187 10.1007/s10661-010-1715-x

[58] Wolch JR, Byrne J, Newell JP (2014) Urban green space, public health, and environmental justice: The challenge of making cities ‘just green enough’. Landscape and Urban Planning.

